# Which variables and determinants influence online food delivery consumption among workers and students? Results from the DELIvery Choice In OUr Society (DELICIOUS) cross-sectional study

**DOI:** 10.3389/fpubh.2023.1326628

**Published:** 2024-01-08

**Authors:** Gianmarco Giacomini, Alessandro Scacchi, Paolo Ragusa, Alessandro Prinzivalli, Heba Safwat Mhmoued Abdo Elhadidy, Maria Michela Gianino

**Affiliations:** Department of Public Health and Pediatric Sciences, School of Medicine, University of Turin, Turin, Italy

**Keywords:** online food delivery, OFD, consumption behavior, risky behaviors, food habits, public health, mindful eating, survey

## Abstract

**Introduction:**

Online food delivery (OFD) applications provide easy access to food, eliminating the need for cooking and meal planning. However, they predominantly promote processed and calorie-dense foods, potentially impacting diet and health. This study aimed to describe the use of OFD services in a sample of Italian workers and students, to explore potential determinants of OFD usage and to assess possible differences in use between these two categories.

**Methods:**

Data were collected through an online survey (convenience sampling) distributed on social media platforms between July 12, 2022, and February 1, 2023. The sample included individuals aged 18 and above, currently residing in Italy. The sample was stratified according to worker/student status. Descriptive analyses were performed on key variables. A multivariable logistic regression analysis was conducted to assess the effects of variables on OFD usage, treated as a dichotomous variable (usage or non-usage).

**Results:**

This study analyzed 3,502 questionnaires (2,594 from workers and 908 from students) after excluding 323 non-student and non-worker responses. Positive associations with OFD use included higher income, remote working, smoking, elevated BMI, depression risk, and impulsive buying among workers, while being female, frequent screen-watching during meals, daily smoking, higher BMI, and impulsive buying were associated with OFD use among students. Factors negatively associated with OFD use included older age (workers and students), living in suburbs (workers and students) or in an outline town (workers), being single (workers and students), adhering to the Mediterranean diet (students), having low health literacy (students), and job dissatisfaction (workers).

**Discussion:**

OFD consumption was associated with some risky behaviors and conditions, such as high BMI or smoking, suggesting that it might be influenced by individual tendencies. Healthier habits, such as physical activity, did not significantly impact OFD usage, especially among workers. Whereas, among students, factors such as low health literacy and better eating habits were associated with less use of OFD, so that they could be more prone to use OFD in a measured way. Further research is needed to better understand potential associations between OFD and risky habits, as well as its role in promoting access to healthy food in underserved areas.

## 1 Introduction

The foodservice industry has undergone profound transformations over the years, mainly driven by technological advancement (1). The internet paved the way for online food delivery (OFD), allowing customers to effortlessly select and order a wide selection of food and beverages from an extensive range of restaurants, which was even more facilitated by the ease and convenience of use of smartphones (2). This paradigm shift is exemplified by a substantial expansion in the geographical radius for food procurement, which increased from a maximum of 1.5 km or 20 min walking distance, to 10 km (3). User-friendly mobile applications further streamlined the ordering process, transforming the dynamics of food acquisition (4, 5). Features such as real-time tracking, estimated delivery fees, and restaurant reviews, have enhanced informed decision-making for customers.

The COVID-19 pandemic, necessitating social restrictions, led to a surge in the demand for food delivery services (6). This heightened demand still persists (7), with expectations that it will endure in the future, regardless of any pandemic or emergency context (8). Presently, the global market for food delivery stands at a valuation of 923.1 billion dollars with 3481.3 million global users. This market has more than quadrupled since 2017, and it is expected to reach 1.45 trillion dollars by 2027 (5). In particular, the Italian OFD market is worth over 5 billion dollars, expecting an annual growth rate of 9.39% from 2023 to 2028 (9).

As convenience and saving time have emerged as essential values in contemporary lifestyles (5, 10, 11), OFD aptly addresses the imperative to procure food with minimal effort, commitment and time investment (12). Fully responding to these needs (13), OFD mobile applications have eliminated barriers associated with traditional purchasing modalities (14). The complexity of home food preparation, encompassing tasks such as cooking, meal planning, dietary considerations, food storage and procurement and subsequent cleanup, is circumvented by the delivery of ready-to-eat meals in disposable containers (15, 16).

Global diets have been influenced over time by a variety of factors, including economic growth, urbanization, increased female labor force participation, and growing international trade in food and other commodities (17, 18). While OFD mobile applications offer unparalleled convenience, the commercial underpinning of these platforms tends to predominantly promote highly palatable, processed, and calorie-dense food. This may exert a deleterious influence on the overall nutritional quality of dietary intake. Despite the wide variety of food options available - including healthy choices - studies underscore the propensity for the most frequently purchased and advertised food to be characterized by large portions high in saturated fat, sugar, and salt (19, 20). Several studies have found adverse health effects associated with increased consumption of unhealthy food, alcohol, and nicotine, linked to the use of OFD, potentially contributing to chronic diseases and mental health risk factors (21). Moreover, concerns regarding OFD use extend to the impact on public health dimensions such as physical activity, sedentariness, and waste production (3).

The literature highlights differences in the utilization of OFD services among students and employed individuals, so that it would be important to assess them separately in order to underline possible differences. Indeed, factors such as age, socio-economic status, and time availability (e.g., work-life balance) influence people’s behavior, including dietary habits: in this sense, financial independence and daily routines can also have a role (22, 23). However, the determinants of OFD services utilization are intricate and multifaceted.

The theoretical framework of this study considers the Health Belief Model (HBM), suggesting that individual behavior is influenced by perceived threats, barriers or benefits perceived for that behavior. For instance, individuals might perceive cooking as stressful and might appreciate the benefits of food delivery (convenience, comfort, and reduced effort), with certain conditions or reasons acting as cue-to-actions (such as feeling depressed and struggling with daily activities). OFD use could also be influenced by an emotion-focused coping mechanism (seeking comfort food), or even depending on motivation to engage in healthy behaviors. Decisions to use OFD can be driven by both reflective (considering health risks) and intuitive - sometimes impulsive - factors, as in a dual-process model (24, 25). Indeed, individuals with higher risk factors or habits (such as high BMI, smoking, low physical activity) might adopt less healthy lifestyles. Moreover, Time-Use Theory suggests that time allocation between activities is based on personal preferences, constraints, and opportunities (26). Diverse daily schedules of workers and students could potentially affect their ability to engage in activities such as cooking, and budget constraints are also important to consider. To the authors’ knowledge, there is a lack of evidence on the determinants of OFD use, especially in the Italian context, which is still understudied and on which our study focused. The research hypotheses of this study are: individuals experiencing depressive symptoms, time constraints, and job dissatisfaction may be less inclined to cook and could rely more on OFD. Those with health risk factors/unhealthy behaviors (e.g., high BMI, low health literacy, unhealthy diet, low physical activity, smoking, etc.) are expected to show increased likelihood of utilizing food delivery services. Being a worker or a student may influence the use of OFD.

Therefore, the aim of this study is to explore potential determinants of OFD use among Italian workers and students. Specifically, we aimed to explore personal, socio-demographic and lifestyle factors influencing the decision to use OFD, and to assess potential differences between the two groups. Indeed, as the increasing consumption of OFD could potentially affect individuals’ dietary habits, it is important to take a closer look at this phenomenon. Characterizing OFD users according to personal, socio-demographic and lifestyle factors is useful to potentially predict how this technology could be used. Moreover, by taking into account the characteristics of users, this phenomenon could be better addressed in nutrition education programmes.

## 2 Materials and methods

### 2.1 Study design and recruitment

A nation-wide cross-sectional study – DELIvery Choice In our Society (DELICIOUS) - was conducted. The Strengthening the Reporting of Observational Studies in Epidemiology (STROBE) (27) for cross-sectional studies was used to report the study. The checklist is available as Supplementary material 1.1. Data were collected between July 12, 2022 and February 1, 2023 through an online survey distributed on social media platforms using the LimeSurvey platform (28). The survey link was spread mainly on Facebook, Instagram and WhatsApp: different types of groups, sites or influencers (e.g., not only food related pages/groups, but also those related to schools, universities, local health units, and others) from various regions of Italy were contacted in order to have as much variability as possible among respondents and thus avoid selection bias.

Inclusion criteria of the study were: being workers or students aged 18 or above; currently living in Italy; being able to read and understand the questionnaires written in Italian; having internet access; having given informed consent. Raosoft® was used to determine that the minimum sample size was 385, considering a 5% margin of error, a 95% confidence level, a 50% response distribution, and the size of the Italian adult population. Participants were enrolled through convenience sampling. Informed consent was required to access the questionnaire. Participation was voluntary, anonymous, and without compensation (to avoid influencing participants). All procedures were in accordance with the 1964 Declaration of Helsinki and its later amendments. The protocol of the study was approved by the Ethics Committee of the University of Turin on July 12, 2022 (Prot. N. 0420932 del 29/07/2022 – [UOR: SI000045 – Classif. III/11]).

### 2.2 Questionnaires

The DELICIOUS survey included the following literature validated self-reported questionnaires, written in Italian:

- Well-being Index 5 (WHO-5) (29): a 5-item questionnaire assessing subjective psychological well-being, screening for depressive symptoms. WHO-5 score ranges from 0 to 25. WHO-5 can be dichotomized according to a validated threshold at 13 points. This is a well-known, widely used and reliable tool in the scientific literature.
- Literature-based adherence score to the Mediterranean diet (Medi-Lite) (30): a 9-item questionnaire to measure adherence to Mediterranean diet. Medi-Lite score goes from 0 to 18, and it has a validated dichotomous threshold at 8.5 points. This is useful to get a thorough insight into dietary habits.
- Single-item physical activity measure (31): a single item questionnaire to measure the weekly frequency of physical activity. Its score goes from 0 to 7. Physical activity is a key lifestyle factor, of which this tool provides a quick yet relevant assessment.
- Single-item literacy screener Italian (SILS-ITA) (32): a single item questionnaire to measure limited reading ability regarding health documents as a proxy of low health literacy. SILS-ITA score goes from 1 to 5, and it has a validated dichotomous threshold at 2. Health literacy is a critical factor influencing health-related decision-making.
- Impulse buying tendency scale (IBS) (33): a 5-item questionnaire to measure the propensity to buy products on impulse. IBS score goes from 1 to 35. Impulsive behavior can therefore influence food choices and dietary habits.
- Work life balance (WLB) (34): a 5-item questionnaire. WLB score goes from 5 to 25. According to our hypotheses, time constraints could influence food choices. Evaluating work-life balance contributes to the understanding of respondents’ perceived time restrictions.
- The Job Satisfaction Single Item (JSSI): a single-item questionnaire to measure the overall job satisfaction (35). The score goes from 1 to 7. According to our hypotheses, job-related factors could influence food choices, and job satisfaction could also impact one’s mental well-being.

In addition, participants were asked about their age, gender, geographic area, city of residence [classified into three groups according to city size and municipal services provided as advised by National Institute of Statistics (36)], relationship status, cohabitation, monthly income [obtained from personal income tax rate per year (37)], educational level, working setting, smoking habit, dietary habit, time spent watching screens while eating (screen time while eating), and information about use of OFD. Self-reported height and weight were asked to calculate individual Body Mass Index (BMI). Whereas a questionnaire was unavailable in Italian, two independent researchers translated them in Italian. Conflicts in the translation were resolved by an additional researcher.

A sample of DELIvery Choice in our Society (DELICIOUS) questionnaire is available as Supplementary material 1.3.

### 2.3 Statistical analysis

In order to highlight possible differences in OFD usage determinants, the sample was stratified according to worker/student status, excluding responses from individuals who were not students or workers. Descriptive analyses were performed for the most prominent variables: frequencies for categorical variables, and medians and interquartile ranges (IQR) for continuous variables were shown, since the normality Shapiro–Wilk test proved non-normal distributions. Data were presented according to the presence or absence of food delivery usage: chi-squared test (for categorical variables) and nonparametric Mann–Whitney test (for continuous variables) were performed.

A multivariable logistic regression analysis was performed to evaluate the effects of variables on food delivery usage: variables were inserted in the model based on previous literature (15, 19, 21), highlighted by collected data and based on researchers’ hypotheses (described in the introduction), as well as variables that were deemed important to control for (e.g., age, gender, city of residence). OFD usage was the outcome variable, and it was used as a dichotomous variable (usage or non-usage). In the multivariable model, the following independent variables were entered: gender, age, city of residence (categorized on the basis of city size and services available), monthly income, working setting, relationship status, being on a diet, following the Mediterranean diet (estimated through the Medi-Lite score), physical activity (according to the single-item physical activity measure), screen time while eating, smoking habit, BMI, risk of being depressed (estimated through the WHO-5 validated scale), health literacy (measured through the SILS-ITA), work life balance (according to the WLB scale), job satisfaction (estimated through the JSSI) and impulse buying tendency (according to the IBS scale). Results were expressed as adjusted odds ratios (AdjOR) and their 95% confidence intervals; the statistical significance threshold was set at 2-tailed *p* < 0.05.

For analytical purposes some variables were aggregated: for example, cohabitation was dichotomized, aggregating living with family members, roommates, and partners, or living alone into living with someone or alone. Physical activity was dichotomized according to the median level of our sample. WHO-5, SILS-ITA, and job satisfaction were dichotomized based on validated threshold values.

To manage missing data, listwise deletion in the logistic regression and pairwise deletion in the descriptive analysis were used.

All analyses were performed with Stata (Version 13.1) software.

## 3 Results

A total of 3,502 questionnaires were included, comprising 2,594 from workers and 908 from students. The median age of the workers was 46 (IQR 38–51) years for those who did not use OFD services and 39 (IQR 34–45) years for those who did use OFD services. Among the students, the respective median ages were 22 (IQR 20–24) and 23 (IQR 21–25) years. Respondents were mostly females both for the workers (90.23%, missing = 4) and for the students (72.86%, missing = 20), mostly from northern regions (70.59 and 91.08%) and main cities of Italy (76.1 and 80.51%). Almost 19.35% of the workers and 47.14% of the students were single at the time of completing the questionnaire, but only 14.8 and 8.59%, respectively, lived alone. More than half of our respondents (54.28 and 57.82%) reported a WHO-5 score above its validated threshold. As our outcome measure, OFD services were widely used in our sample: 77.37% for the workers and 79.07% for the students. Several personal (working setting, WHO-5, IBS, SILS-ITA, job satisfaction for workers; relationship status, WHO-5, IBS, SILS-ITA, screen time while eating for students), socio-demographic (age, city of residence, monthly income, educational level for workers; gender, city of residence for students) and lifestyle factors (smoking habit for workers; smoking habit, BMI, Medi-Lite for students) were significantly related with OFD use according to the chi-squared tests and Mann–Whitney tests. The detailed description of the sample is shown in Table 1.

**Table 1:** Participants according to their food delivery consumption.

		Workers (*n* = 2,594)			Students (*n* = 908)		
		Delivery usage		*p*	Delivery usage		*p*
		No (*n* = 587)	Yes (*n* = 2007, 77.37%)		No (*n* = 190)	Yes (*n* = 718, 79.07%)	
Age median (IQR)		46 (38–51)	39 (34–45)	**<0.001**	22 (20–24)	23 (21–25)	0.108
Gender *n* (%)	Males	65 (11.09%)	188 (9.38%)	0.220	64 (34.04%)	177 (25.29%)	**0.017**
	Females	521 (88.91%)	1816 (90.62%)		124 (65.96%)	523 (74.71%)	
Geographic area *n* (%)	North	421 (71.72%)	1,410 (70.25%)	0.282	177 (93.16%)	650 (90.53%)	0.523
	Centre	84 (14.31%)	340 (16.94%)		7 (3.68%)	35(4.87%)	
	South and Islands	82 (13.97%)	257 (12.81%)		6 (3.16%)	33 (4.60%)	
City of residence *n* (%)	Main cities	367 (62.52)	1,607 (80.07)	**<0.001**	111 (58.42)	620 (86.35)	**<0.001**
	Suburbs	203 (34.58)	386 (19.23)		76 (40.00)	95 (13.23)	
	Outlying towns	17 (2.90)	14 (0.70)		3 (1.58)	3 (0.42)	
Sentimental status *n* (%)	Not single	463 (78.88%)	1,629 (81.17%)	0.217	77 (40.53%)	403 (56.13%)	**<0.001**
	Single	124 (21.12%)	378 (18.83%)		113 (59.47%)	315 (43.87%)	
Cohabitation *n* (%)	No	498 (84.84%)	1712 (85.30%)	0.781	179 (94.21%)	651 (90.67%)	0.121
	Yes	89 (15.16%)	295 (14.70%)		11 (5.79%)	67 (9.33%)	
Monthly income* *n* (%)	<2,333	495 (84.33%)	1,593 (79.37%)	**0.027**	183 (96.32%)	697 (97.08%)	0.148
	2,333–4,583	77 (13.12%)	339 (16.89%)		2 (1.05%)	14 (1.95%)	
	>4,583	15 (2.56%)	75 (3.74%)		5 (2.63%)	7 (0.97%)	
Educational level *n* (%)	Below secondary	280 (47.70%)	636 (31.69%)	**<0.001**				Postsecondary	307 (52.30%)	1,371 (68.31%)			
Working setting *n* (%)	Mostly/fully in presence	467 (87.03%)	1,694 (81.64%)		**0.002**				Mostly/fully remotely	69 (12.97%)	381 (18.36%)			
Smoke habit	Never	491 (83.65%)	1,490 (74.24%)	**<0.001**	161 (84.74%)	495 (68.94%)	**<0.001**	Occasionally	36 (6.13%)	177 (8.82%)	16 (8.42%)	93 (12.95%)	Everyday	60 (10.22%)	340 (16.94%)	13 (6.84%)	130 (18.11%)
BMI median (IQR)		23.44 (21.09–26.58)	23.66 (21.23–26.93)		0.151	20.95 (19.53–23.12)		21.86 (19.95–24.31)	**0.003**
Physical activity *n* (%)	< two times per week	263 (46.80%)	903 (46.31%)		0.838	45 (24.32%)		214 (30.31%)	0.110
	≥ two times per week	299 (53.20%)	1,047 (53.69%)	140 (75.68%)		492 (69.69%)			
Being on a diet *n* (%)	No	409 (69.68%)	1,473 (73.39%)	0.076	141 (74.21%)	530 (73.82%)	0.912
	Yes	178 (30.32%)	534 (26.61%)		49 (25.79%)	188 (26.18%)	
WHO-5 *n* (%)	At risk	308 (52.47%)	878 (43.75%)	**<0.001**	92 (48.82%)	291(40.53%)	**0.050**
	Not at risk	279 (47.53%)	1,129 (56.25%)		98 (51.58%)	427 (59.47%)	
IBS score median (IQR)		8 (4–12)	10 (6–14)	**<0.001**	5.5 (3–9)	8 (5–11)	**<0.001**
SILS-ITA *n* (%)	High HL	519 (88.42%)	1841 (91.73%)	**0.014**	154 (81.05%)	628 (87.47%)	**0.023**
	Low HL	68 (11.58%)	234 (8.27%)		36 (18.95%)	90 (12.53%)	
Screen time while eating *n* (%)	Never/seldom	217 (36.97%)	659 (32.84%)	0.063	69 (36.32%)	126 (17.55%)	**<0.001**
	Often/always	370 (63.03%)	1,348 (67.16%)		121(63.68%)	592 (82.45%)	
Medi-Lite score median (IQR)		10 (8–11)	10 (8–11)	0.387	10 (9–12)	9 (8–11)	**<0.001**
WLB score median (IQR)		12 (9–14)	12 (8–15)	0.674			
Job satisfaction *n* (%)	Satisfied	344 (67.06%)	1,324 (71.80%)	**0.037**			
	Unsatisfied	169 (32.94%)	520 (28.20%)				

IQR, Interquartile Range; n, number; BMI, Body Mass Index; WHO-5, 5-item World Health Organization Well-Being Index questionnaire; SILS-ITA, Italian Single-Item Literacy Screener; HL, Health Literacy; WLB, Work Life Balance; IBS, Impulse Buying Scale. To enhance readability, *p*-values <0.05 are shown bolded. *Currency: euros.

Workers multivariable logistic regression analysis - based on 2,270 individuals due to listwise deletion of missing data - showed some factors significantly associated with OFD consumption. In particular, having a higher income (OR: 1.99; CI: 1.01–3.90), carrying out the work activity mostly or completely remotely (OR: 1.37; CI: 1.01–1.85), being an occasional (OR: 1.63; CI:1.05–2.55) or daily (OR:1.93; CI: 1.37–2.73) smoker, having a higher BMI (OR: 1.03; CI: 1.00–1.05), being at risk for depression (OR: 1.41; CI: 1.12–1.78), and tending to impulsive buying (OR: 1.05; CI: 1.03–1.07) were positively associated with the use of OFD. On the other hand, older age (OR: 0.93; CI: 0.92–0.95), living in a suburb (OR: 0.44; CI: 0.35–0.56) or in an outlying town (OR: 0.17; CI: 0.08–0.40), being single (OR: 0.73; CI: 0.55–0.95), and being dissatisfied with job (OR:0.78; CI: 0.61–0.99) were negatively associated with OFD consumption.

Multivariable logistic regression analysis in the student sample - based on 863 individuals due to listwise deletion of missing data - showed that being female (OR: 1.55; CI: 1.02–2.34), watching a screen while eating (OR: 2.76; CI: 1.82–4.19), being a daily smoker (OR:2.94; CI: 1.50–5.77), having a higher BMI (OR: 1.07; CI: 1.01–1.13), and tending to impulsive buying (OR: 1.09; CI: 1.04–1.14) were positively associated with the use of OFD. On the other hand, older age (OR: 0.95; CI: 0.92–0.99), living in a suburb (OR: 0.20; CI: 0.13–0.30), being single (OR: 0.49; CI: 0.33–0.72), having a higher adherence to the Mediterranean diet (OR: 0.89; CI: 0.81–0.96), and having a low level of health literacy (OR: 0.58; CI: 0.35–0.97) were negatively associated with OFD consumption.

Multivariable logistic regression analysis results are shown in Table 2, while full data (including also Standard Error and z-score) can be found as Supplementary material 1.2.

**Table 2:** Multivariable logistic regression analysis.

		Workers (*n* = 2,270)			Students (*n* = 863)		
	**Delivery usage**	**OR**	**P > z**	**[95% CI]**	**OR**	**P > z**	**[95% CI]**
Gender	Male	1			1		
	Female	1.33	0.131	0.92–1.91	1.55	**0.038**	1.02–2.34
Age		0.93	**0.000**	0.92–0.95	0.95	**0.013**	0.92–0.99
City of residence	Main cities	1			1		
	Suburb	0.44	**0.000**	0.35–0.56	0.20	**0.000**	0.13–0.30
	Outlying towns	0.17	**0.000**	0.08–0.40	0.21	0.077	0.04–1.19
Monthly income*	<2,333	1					
	2,333–4,583	1.34	0.066	0.98–1.82	1.28	0.761	0.26–6.20
	>4,583	1.99	**0.046**	1.01–3.90	0.54	0.381	0.14–2.14
Working setting	Mostly/fully in presence	1					
	Mostly/fully remotely	1.37	**0.045**	1.01–1.85			
Sentimental status	Not single	1			1		
	Single	0.73	**0.021**	0.55–0.95	0.49	**0.000**	0.33–0.72
Being on a diet	No	1			1		
	Yes	0.99	0.922	0.77–1.27	1.26	0.317	0.80–1.97
Medi-Lite score		1.01	0.642	0.96–1.06	0.89	**0.005**	0.81–0.96
Physical activity	<2 times/week	1			1		
	≥2 times/week	0.98	0.835	0.78–1.22	0.99	0.967	0.64–1.52
Screen time while eating	Never/seldom	1			1		
	Often/always	1.01	0.910	1.05–2.55	2.76	**0.000**	1.82–4.19
Smoke habit	Never	1			1		
	Occasionally	1.63	**0.031**	1.05–2.55	1.68	0.114	0.88–3.20
	Everyday	1.93	**0.000**	1.37–2.73	2.94	**0.002**	1.50–5.77
BMI		1.03	**0.020**	1.00–1.05	1.07	**0.016**	1.01–1.13
WHO-5	Not at risk	1			1		
	At risk	1.41	**0.003**	1.12–1.78	1.04	0.827	0.71–1.53
SILS-ITA	Good HL	1			1		
	Bad HL	0.76	0.126	0.53–1.08	0.58	**0.037**	0.35–0.97
WLB score		1.01	0.582	0.98–1.04			
Job satisfaction	Satisfied	1					
	Unsatisfied	0.78	**0.043**	0.61–0.99			
IBS score		1.05	**0.000**	1.03–1.07	1.09	**0.000**	1.04–1.14

Outcome: food delivery consumption. OR, Odds Ratio; CI, Confidence Interval; Medi-Lite: Literature-based adherence score to Mediterranean diet; BMI, Body Mass Index; WHO-5, 5-item World Health Organization Well-Being Index questionnaire; SILS-ITA, Italian Single-Item Literacy Screener; HL, Health Literacy; WLB, Work Life Balance; IBS, Impulse Buying Scale. To enhance readability, *p*-values <0.05 are shown bolded. *Currency: euros.

## 4 Discussion

This study aimed to assess the use of OFD in a sample of Italian workers and students, and the potential factors influencing its usage. OFD services were widely used in our sample in the last 12 months: 77.37% utilization among workers and 79.07% among students.

Main results in the workers sample showed that having a higher income, working mostly or fully remotely, being an occasional or daily smoker, having a higher BMI, being at risk for depression, and having a tendency to impulsive buying, were positively associated with the use of OFD. Instead, older age, living in a suburb or in an outlying town, being single, and being job dissatisfied were negatively associated with OFD consumption.

In the student sample being female, watching a screen while eating, being a daily smoker, higher BMI, and impulsive buying were positively associated with OFD use. Instead, being older, living in a suburb, being single, having a higher adherence to Mediterranean diet, and having a low level of health literacy were negatively associated with OFD consumption.

In general, our results reported a higher prevalence of OFD users compared to market analyses, which predicted a user penetration of 25% in Italy (2023 data) (9). Possibly, the digital format of the questionnaire favored technology-savvy respondents, as also reported in a previous similar study (23).

Young age was significantly related to OFD use, consistently with previous research (23). This result is in line with young people’s tendency to be more accustomed to technology, to be time-starved and convenience-seeking (38–40). Moreover, young adults, especially those living alone, may lack confidence in their cooking abilities, whereas food delivery applications may provide an easy and convenient alternative (22). On the other hand, older individuals could be less acquainted with new technologies, and they could be more prone to use conventional food ordering methods (23, 41). Loyalty and personal affection as a customer are key elements for repurchase (23, 42), and older people may not perceive some features of OFD services as affective elements, even if they are capable of positively influencing the customers experience (e.g., personalization, order customization and information availability) (42, 43).

Among workers remote work correlated positively with OFD use, emphasizing the time-saving convenience and practicality of ordering food online while working remotely (23, 44, 45).

Surprisingly, lower levels of job satisfaction were negatively linked to OFD use. Although this finding may be related to poor mental health, it is difficult to interpret and requires further, more specific research.

Among students, eating in front of a screen was associated with OFD use. Eating while watching a screen is known to be associated with sedentariness and mindless eating activity, that could potentially lead to reduced awareness of one’s own dietary choices, affecting overall health and nutrition (46).

Female students were more likely to be OFD users. This is in contrast with some previous studies and market analyses (42, 47), but our sample was skewed towards the female gender.

In both groups, we found a higher usage of OFD in main cities (those with more health, education, and transport services). This could be due to the wide range of dining and OFD convenient options available in these contexts (23, 45).

Incomes over 4,583 euros per month were positively related with OFD use among workers. This finding contrasts with market data, which suggest a correlation between lower incomes and increased OFD usage (40).

In both groups, relationship status influenced OFD usage, with single individuals being less likely to use OFD, possibly because of the convenience of sharing a meal together with considerable reduction of costs (43).

BMI was positively associated with OFD consumption both in workers and students, consistently with previous research (23, 48). It is known that eating meals prepared away from home, especially considering fast-food options, is related to higher food consumption, and eventually increased risk of being overweight (49).

Adherence to the Mediterranean diet is associated with the non-utilization of OFD services among students, who could be more careful about making healthier choices about their food habits. Indeed, OFD allows ordering from a plethora of different food outlets also including healthy choices.

Unexpectedly, among students, lower levels of health literacy are linked to the non-utilization of OFD services. Health literacy is crucial for a properly informed health-related decision-making: low health literacy is associated with various health risks, including inadequate self-care behaviors, reduced utilization of preventive health services, higher rates of chronic illness, and diminished physical and mental health (32, 50, 51). Although this finding may be related to poorer use of technology by those with lower health literacy, more in-depth studies are needed.

In both groups, impulse buying tendencies were significantly associated with OFD consumption. OFD allows easy access to a variety of food options and could encourage impulsiveness. Given that impulsivity has been correlated with higher food intake and unhealthier food choices (49), impulsive use of OFD services could potentially lead to poorer nutrition choices.

Experiencing symptoms suggestive of depression led workers to be more prone to OFD usage. This phenomenon can be understood within the broader context of emotional eating, where individuals turn to food as a means to cope with negative emotions and psychological distress. Comfort foods, often high in calories and easily accessible through OFD services, can provide temporary relief from depressive symptoms (52). Previous research reports that OFD could make comfort food more available to those experiencing psychological distress (53). The convenience and anonymity of ordering food online may also appeal to individuals who are experiencing low mood, as it requires minimal social interaction and effort, but specific causes of workers’ depressive symptoms should be further assessed.

Smoking was associated with OFD consumption in students and workers. Previous literature evidenced that smokers are known to have a less healthy diet with more food craving, especially for food high in fats. This habit appears to be related to several facts, both neurological and psychological, such as altered fat-taste perception (54). As smokers tend to prefer flavorful food, often on impulse, the large, fast, and varied offers of OFD could meet these needs.

### 4.1 Limitations

This study has some limitations. Firstly, the cross-sectional design precluded conclusions about the direction of the relationships, so that future longitudinal studies will be needed to better understand the causality links. Secondly, as self-administered questionnaires have been used, recall bias could be present. Despite the measures employed, common method bias could be present. Lastly, convenience sampling could have limited the variety of the sample potentially lowering the external validity of the results.

### 4.2 Conclusion

This study investigated OFD consumption among students and workers, examining its correlation with various personal, socio-demographic and lifestyle factors.

OFD consumption exhibited distinct patterns of association with some differences between students and workers.

In conclusion, our study highlights the need for targeted considerations addressing the distinct habits influencing OFD use of students and workers. From a theoretical point of view, it would be important to further assess OFD use in different groups of people, searching for specific determinants of its usage. Eventually, OFD could be a promising tool for potential diet quality improvement interventions. In practical terms, it would be important to consider OFD when implementing educational policies that promote healthy lifestyles, emphasize good nutrition and the ability to recognize quality food. Educational initiatives should extend to promoting conscious and informed decision-making in food consumption. Indeed, the use of OFD is not negative *a priori* and it seems to be a tool whose use varies greatly depending on the user’s habits. Therefore, educational efforts should focus on increasing awareness and understanding the validity of one’s choices, rather than discouraging OFD use. If subsequent research will show associations between OFD use and poor diet, the emphasis on nutrition education will become even more important. While nutrition education programmes are often provided during childhood and school age, such programmes are offered to workers much less frequently, so that they could risk neglecting their dietary habits. When organizing interventions on healthy eating and lifestyle, it would be important to take into account the specific characteristics of workers, such as the working setting and job satisfaction, that should be further studied.

## Data availability statement

The raw data supporting the conclusions of this article will be made available by the authors, without undue reservation.

## Ethics statement

The studies involving humans were approved by Comitato bioetico di Ateneo - Università degli Studi di Torino. The studies were conducted in accordance with the local legislation and institutional requirements. The participants provided their written informed consent to participate in this study.

## Author contributions

GG: Conceptualization, Investigation, Writing – review & editing, Data curation, Formal analysis, Methodology, Supervision, Writing – original draft. AS: Conceptualization, Investigation, Methodology, Writing – original draft. PR: Conceptualization, Investigation, Writing – original draft, Writing – review & editing, Data curation, Formal analysis, Methodology, Visualization. AP: Conceptualization, Investigation, Writing – review & editing, Visualization. HE: Visualization, Writing – review & editing. MG: Conceptualization, Data curation, Investigation, Methodology, Project administration, Supervision, Writing – original draft, Writing – review & editing.

## References

[1] Meemken EM Bellemare MF Reardon T Vargas CM. Research and policy for the food-delivery revolution. Science. (2022) 377:810–3. doi: 10.1126/science.abo2182, PMID: 35981021

[2] Chowdhury R. Impact of perceived convenience, service quality and security on consumers’ behavioural intention towards online food delivery services: the role of attitude as mediator. SN Bus Econ. (2023) 3:29. doi: 10.1007/s43546-023-00422-7, PMID: 36643187 PMC9825085

[3] Maimaiti M Zhao X Jia M Ru Y Zhu S. How we eat determines what we become: opportunities and challenges brought by food delivery industry in a changing world in China. Eur J Clin Nutr. (2018) 72:1282–6. doi: 10.1038/s41430-018-0191-1, PMID: 30185849

[4] Stephens J Miller H Militello L. Food delivery apps and the negative health impacts for Americans. Front Nutr. (2020) 7:7. Available from: doi: 10.3389/fnut.2020.00014 32154262 PMC7044187

[5] Statista. Online food delivery – market data analysis & forecast | Statista. (2023). Available at: https://www.statista.com/study/40457/food-delivery/

[6] Ferrante P. The first 2 years of COVID-19 in Italy: incidence, lethality, and health policies. Front Public Health. (2022) 10:986743. doi: 10.3389/fpubh.2022.986743, PMID: 36388357 PMC9664068

[7] Li Y Yao P Osman S Zainudin N Sabri MF. A thematic review on using food delivery services during the pandemic: insights for the post-COVID-19 era. Int J Environ Res Public Health. (2022) 19:15267. doi: 10.3390/ijerph192215267, PMID: 36429983 PMC9690128

[8] Roberts C Young L Johanson M. Theory of dining. Journal of Hospitality & Tourism Research. (2022) 46:1574–95. doi: 10.1177/1096348020988338

[9] Statista. Meal delivery - Italy | Statista market forecast. [cited 2023 Aug 8]. Available at: https://www.statista.com/outlook/dmo/online-food-delivery/meal-delivery/italy?currency=EUR

[10] Jilcott Pitts SB Ng SW Blitstein JL Gustafson A Kelley CJ Pandya S. Perceived advantages and disadvantages of online grocery shopping among special supplemental nutrition program for women, infants, and children (WIC) participants in eastern North Carolina. Curr Dev Nutr. (2020) 4:nzaa076. doi: 10.1093/cdn/nzaa076, PMID: 32399508 PMC7204786

[11] Blitstein JL Frentz F Jilcott Pitts SB. A mixed-method examination of reported benefits of online grocery shopping in the United States and Germany: is health a factor? J Food Prod Mark. (2020) 26:212–24. doi: 10.1080/10454446.2020.1754313

[12] Elms J de Kervenoael R Hallsworth A. Internet or store? An ethnographic study of consumers’ internet and store-based grocery shopping practices. J Retail Consum Serv. (2016) 32:234–43. doi: 10.1016/j.jretconser.2016.07.002

[13] Belk RW. Qualitative consumer research. Bingley, UK: Emerald Publishing (2017).

[14] Etumnu CE Foster KA Widmar NO Lusk JL Ortega DL. Drivers of online grocery shopping. (2019) Available at: https://ageconsearch.umn.edu/record/290858/files/Abstracts_19_05_14_13_09_26_99__128_210_107_129_0.pdf

[15] Larson NI Nelson MC Neumark-Sztainer D Story M Hannan PJ. Making time for meals: meal structure and associations with dietary intake in young adults. J Am Diet Assoc. (2009) 109:72–9. doi: 10.1016/j.jada.2008.10.017 19103325

[16] Short F. Domestic cooking skills: what are they? Journal of the Home Economics Institute of Australia.. (2003) 10:13–22. doi: 10.3316/aeipt.133139

[17] Reardon T Heiman A Lu L Nuthalapati CSR Vos R Zilberman D. “Pivoting” by food industry firms to cope with COVID-19 in developing regions: E-commerce and “copivoting” delivery intermediaries. Agric Econ. (2021) 52:459–75. doi: 10.1111/agec.12631, PMID: 34230730 PMC8250822

[18] Popkin BM Reardon T. Obesity and the food system transformation in Latin America. Obes Rev. (2018) 19:1028–64. doi: 10.1111/obr.12694, PMID: 29691969 PMC6103889

[19] Lake AA. Neighbourhood food environments: food choice, foodscapes and planning for health. Proc Nutr Soc. (2018) 77:239–46. doi: 10.1017/S0029665118000022, PMID: 29493482

[20] Partridge SR Gibson AA Roy R Malloy JA Raeside R Jia SS. Junk food on demand: a cross-sectional analysis of the nutritional quality of popular online food delivery outlets in Australia and New Zealand. Nutrients. (2020) 12:3107. doi: 10.3390/nu12103107, PMID: 33053705 PMC7601596

[21] World Health Organization. Regional Office for Europe. Digital food environments: factsheet. (2021) Available at: https://apps.who.int/iris/handle/10665/342072

[22] Buettner SA Pasch KE Poulos NS. Factors associated with food delivery app use among Young adults. J Community Health. (2023) 6:1–7. doi: 10.1007/s10900-023-01229-1 PMC10163566 37148460

[23] Keeble M Adams J Sacks G Vanderlee L White CM Hammond D. Use of online food delivery services to order food prepared away-from-home and associated sociodemographic characteristics: a cross-sectional, multi-country analysis. Int J Environ Res Public Health. (2020) 17:5190. doi: 10.3390/ijerph17145190 32709148 PMC7400536

[24] Bellini-Leite SC. Dual process theory: embodied and predictive; symbolic and classical. Front Psychol. (2022) 13:13. doi: 10.3389/fpsyg.2022.805386, PMID: 35386885 PMC8979207

[25] Leshem R. Using dual process models to examine impulsivity throughout neural maturation. Dev Neuropsychol. (2016) 41:125–43. doi: 10.1080/87565641.2016.1178266, PMID: 27186976

[26] Becker GS. A theory of the allocation of time. Econ J. (1965) 75:493–517. doi: 10.2307/2228949

[27] von Elm E Altman DG Egger M Pocock SJ Gøtzsche PC Vandenbroucke JP. The strengthening the reporting of observational studies in epidemiology (STROBE) statement: guidelines for reporting observational studies. J Clin Epidemiol. (2008) 61:344–9. doi: 10.1016/j.jclinepi.2007.11.008 18313558

[28] LimeSurvey GH. Hamburg. Germany. Available at: http://www.limesurvey.org

[29] Topp CW Østergaard SD Søndergaard S Bech P. The WHO-5 well-being index: a systematic review of the literature. PPS. (2015) 84:167–76. doi: 10.1159/000376585 25831962

[30] Sofi F Dinu M Pagliai G Marcucci R Casini A. Validation of a literature-based adherence score to Mediterranean diet: the MEDI-LITE score. Int J Food Sci Nutr. (2017) 68:757–62. doi: 10.1080/09637486.2017.1287884, PMID: 28276908

[31] Wanner M Probst-Hensch N Kriemler S Meier F Bauman A Martin BW. What physical activity surveillance needs: validity of a single-item questionnaire. Br J Sports Med. (2014) 48:1570–6. doi: 10.1136/bjsports-2012-092122, PMID: 23770662

[32] Bonaccorsi G Grazzini M Pieri L Santomauro F Ciancio M Lorini C. Assessment of Health Literacy and validation of single-item literacy screener (SILS) in a sample of Italian people. Ann Ist Super Sanita (2017) 53:205–212. doi: 10.4415/ANN_17_03_05 28956799

[33] Weun S Jones MA Beatty SE. Development and validation of the impulse buying tendency scale. Psychol Rep. (1998) 82:1123. doi: 10.2466/pr0.1998.82.3c.1123 9709520

[34] Brough P Timms C O’Driscoll MP Kalliath T Siu O Sit C Lo D. Work–life balance: a longitudinal evaluation of a new measure across Australia and New Zealand workers. The International Journal of Human Resource Management.. (2014) 25:2724–2744. doi: 10.1080/09585192.2014.899262

[35] Dolbier C Webster J Mccalister K Mallon M Steinhardt M. Reliability and validity of a single-item measure of job satisfaction. American journal of health promotion: AJHP. (2005) 19:194–8. doi: 10.4278/0890-1171-19.3.194, PMID: 15693347

[36] Istituto Nazionale di Statistica. La geografia delle aree interne nel 2020 - vasti territori tra potenzialità e debolezze. (2022). Available at: https://www.istat.it/it/archivio/273176

[37] Agenzia delle Entrate. Imposta sul reddito delle persone fisiche (Irpef) - Aliquote e calcolo dell’Irpef. Available at: https://www.agenziaentrate.gov.it/portale/imposta-sul-reddito-delle-persone-fisiche-irpef-/aliquote-e-calcolo-dell-irpef

[38] Cheng A. Forbes. Millennials Are Ordering More Food Delivery, But Are They Killing The Kitchen, Too? (2018). Available at: https://www.forbes.com/sites/andriacheng/2018/06/26/millennials-are-ordering-food-for-delivery-more-but-are-they-killing-the-kitchen-too/

[39] DoorDash. The rise of food delivery services: why consumers order in | DoorDash for merchants. (2020). Available at: https://get.doordash.com/en-us/blog/rise-in-food-delivery-and-why-it-is-popular

[40] Zion A Hollmann T. Food delivery apps: usage and demographics. Zion & Zion. (2019). Available at: https://www.zionandzion.com/research/food-delivery-apps-usage-and-demographics-winners-losers-and-laggards/

[41] Amis A Chawla M Tulpule D. A comparative study of online food delivery START-ups in the food industry. International Journal of Current Research. (2021) 13:17540–9. doi: 10.24941/ijcr.41407.05.2021

[42] Riaz H Davidaviciene V Ahmed H Meidute-Kavaliauskiene I. Optimizing customer repurchase intention through cognitive and affective experience: an insight of food delivery applications. Sustainability. (2022) 14:12936. doi: 10.3390/su141912936

[43] Cho M Bonn MA Li J. Differences in perceptions about food delivery apps between single-person and multi-person households. Int J Hosp Manag. (2019) 77:108–16. doi: 10.1016/j.ijhm.2018.06.019

[44] Robson SM Crosby LE Stark LJ. Eating dinner away from home: perspectives of middle-to high-income parents. Appetite. (2016) 96:147–53. doi: 10.1016/j.appet.2015.09.019, PMID: 26386299 PMC4684743

[45] Keeble M Adams J Burgoine T. Investigating experiences of frequent online food delivery service use: a qualitative study in UK adults. BMC Public Health. (2022) 22:1365. doi: 10.1186/s12889-022-13721-9, PMID: 35842625 PMC9287535

[46] Adate A Shahi S Alharbi R Sen S Gao Y Katsaggelos AK. Detecting screen presence with activity-oriented RGB camera in egocentric videos. Proc IEEE Int Conf Pervasive Comput Commun Workshops. (2022) 2022:403–8. doi: 10.1109/percomworkshops53856.2022.9767433, PMID: 36448975 PMC9704366

[47] Just Eat. justeat.it. (2020). Tutti i numeri del quarto Osservatorio Just Eat. Available at: https://www.justeat.it/blog/progetti-e-impegno-in-italia/il-quarto-osservatorio-just-eat

[48] Albalawi AA Hambly C Speakman JR. Consumption of takeaway and delivery meals is associated with increased BMI and percent fat among UK biobank participants. Am J Clin Nutr. (2022) 116:173–88. doi: 10.1093/ajcn/nqac078, PMID: 35681260 PMC9257477

[49] Sarmugam R Worsley A. Dietary Behaviours, impulsivity and food involvement: identification of three consumer segments. Nutrients. (2015) 7:8036–57. doi: 10.3390/nu7095379, PMID: 26393649 PMC4586574

[50] Jayasinghe UW Harris MF Parker SM Litt J van Driel M Mazza D. The impact of health literacy and life style risk factors on health-related quality of life of Australian patients. Health Qual Life Outcomes. (2016) 14:68. doi: 10.1186/s12955-016-0471-1 27142865 PMC4855442

[51] Shahid R Shoker M Chu LM Frehlick R Ward H Pahwa P. Impact of low health literacy on patients’ health outcomes: a multicenter cohort study. BMC Health Serv Res. (2022) 22:1148. doi: 10.1186/s12913-022-08527-9, PMID: 36096793 PMC9465902

[52] van Strien T Gibson EL Baños R Cebolla A Winkens LHH. Is comfort food actually comforting for emotional eaters? A (moderated) mediation analysis. Physiol Behav. (2019) 211:112671. doi: 10.1016/j.physbeh.2019.112671, PMID: 31484047

[53] Finch LE Tomiyama AJ. Comfort eating, psychological stress, and depressive symptoms in young adult women. Appetite. (2015) 95:239–44. doi: 10.1016/j.appet.2015.07.017, PMID: 26192221

[54] Chao AM White MA Grilo CM Sinha R. Examining the effects of cigarette smoking on food cravings and intake, depressive symptoms, and stress. Eat Behav. (2017) 24:61–5. doi: 10.1016/j.eatbeh.2016.12.009, PMID: 28038436 PMC5269575

