# Supplementary material for: Which variables and determinants influence online food delivery consumption among workers and students? Results from the DELIvery Choice In OUr Society (DELICIOUS) cross-sectional study
Source: Front Public Health. 2024 Jan 8;11:1326628. doi: 10.3389/fpubh.2023.1326628 (PMC10801387; doi:10.3389/fpubh.2023.1326628)
Supplement: Supplementary file 1 [file Table_1.docx]

***Supplementary Material***

**Supplementary Material 1.1 - STROBE Statement - Checklist of items that should be included in reports of *cross-sectional studies*.**

|  | **Item No** | **Recommendation** | **Page** |
| --- | --- | --- | --- |
| **Title and abstract** | 1 | (*a*) Indicate the study’s design with a commonly used term in the title or the abstract | 1 |
|  |  | (*b*) Provide in the abstract an informative and balanced summary of what was done and what was found | 1 |
| **Introduction** | | | |
| Background/rationale | 2 | Explain the scientific background and rationale for the investigation being reported | From2 to 3 |
| Objectives | 3 | State specific objectives, including any prespecified hypotheses | From 2 to 3 |
| **Methods** | | | |
| Study design | 4 | Present key elements of study design early in the paper | 3 |
| Setting | 5 | Describe the setting, locations, and relevant dates, including periods of recruitment, exposure, follow-up, and data collection | 3 |
| Participants | 6 | (*a*) Give the eligibility criteria, and the sources and methods of selection of participants | 3 |
| Variables | 7 | Clearly define all outcomes, exposures, predictors, potential confounders, and effect modifiers. Give diagnostic criteria, if applicable | 3 |
| Data sources/ measurement | 8* | For each variable of interest, give sources of data and details of methods of assessment (measurement). Describe comparability of assessment methods if there is more than one group | 3 |
| Bias | 9 | Describe any efforts to address potential sources of bias | 3 |
| Study size | 10 | Explain how the study size was arrived at | 3 |
| Quantitative variables | 11 | Explain how quantitative variables were handled in the analyses. If applicable, describe which groupings were chosen and why | 3 |
| Statistical methods | 12 | (*a*) Describe all statistical methods, including those used to control for confounding | From 3 to 4 |
|  |  | (*b*) Describe any methods used to examine subgroups and interactions | / |
|  |  | (*c*) Explain how missing data were addressed | 4 |
|  |  | (*d*) If applicable, describe analytical methods taking account of sampling strategy | From 3 to 4 |
|  |  | (*e*) Describe any sensitivity analyses | / |
| **Results** | | | |
| Participants | 13* | (a) Report numbers of individuals at each stage of study—eg numbers potentially eligible, examined for eligibility, confirmed eligible, included in the study, completing follow-up, and analysed | 4 |
|  |  | (b) Give reasons for non-participation at each stage | 4 |
|  |  | (c) Consider use of a flow diagram | / |
| Descriptive data | 14* | (a) Give characteristics of study participants (eg demographic, clinical, social) and information on exposures and potential confounders | From 4 to 5 |
|  |  | (b) Indicate number of participants with missing data for each variable of interest | 4 |
| Outcome data | 15* | Report numbers of outcome events or summary measures | From 4 to 6 |
| Main results | 16 | (*a*) Give unadjusted estimates and, if applicable, confounder-adjusted estimates and their precision (eg, 95% confidence interval). Make clear which confounders were adjusted for and why they were included | From 4 to 6 |
|  |  | (*b*) Report category boundaries when continuous variables were categorized | From 4 to 6 |
|  |  | (*c*) If relevant, consider translating estimates of relative risk into absolute risk for a meaningful time period | / |
| Other analyses | 17 | Report other analyses done—eg analyses of subgroups and interactions, and sensitivity analyses | / |
| **Discussion** | | | |
| Key results | 18 | Summarise key results with reference to study objectives | From 4 to 7 |
| Limitations | 19 | Discuss limitations of the study, taking into account sources of potential bias or imprecision. Discuss both direction and magnitude of any potential bias | 7 |
| Interpretation | 20 | Give a cautious overall interpretation of results considering objectives, limitations, multiplicity of analyses, results from similar studies, and other relevant evidence | From 4 to 7 |
| Generalisability | 21 | Discuss the generalisability (external validity) of the study results | From 4 to 7 |
| **Other information** | | | |
| Funding | 22 | Give the source of funding and the role of the funders for the present study and, if applicable, for the original study on which the present article is based | 8 |

*Give information separately for exposed and unexposed groups.

**Note:** An Explanation and Elaboration article discusses each checklist item and gives methodological background and published examples of transparent reporting. The STROBE checklist is best used in conjunction with this article (freely available on the Web sites of PLoS Medicine at http://www.plosmedicine.org/, Annals of Internal Medicine at http://www.annals.org/, and Epidemiology at http://www.epidem.com/). Information on the STROBE Initiative is available at www.strobe-statement.org.

**Supplementary Material 1.2 - Multivariable logistic regression analysis. Outcome: food delivery consumption.**

|  | **Workers(n=2270)** | | | | | **Students (n=863)** | | | | |
| --- | --- | --- | --- | --- | --- | --- | --- | --- | --- | --- |
| **Delivery usage** | **OR** | **SE** | **z** | **P>z** | **[95% CI]** | **OR** | **SE** | **z** | **P>z** | **[95% CI]** |
| **Gender** |  |  |  |  |  |  |  |  |  |  |
| Male | 1 |  |  |  |  | 1 |  |  |  |  |
| Female | 1.33 | .248 | 1.51 | .131 | .92-1.91 | 1.55 | .327 | 2.07 | **.038** | 1.02-2.34 |
| **Age** | .93 | .006 | -10.58 | **.000** | .92-.95 | .95 | .019 | -2.48 | **.013** | .92-.99 |
| **City of residence** |  |  |  |  |  |  |  |  |  |  |
| Main cities | 1 |  |  |  |  | 1 |  |  |  |  |
| Suburb | .44 | .053 | -6.78 | **.000** | .35-.56 | .20 | .042 | -7.68 | **.000** | .13-.30 |
| Outlying towns | .17 | .074 | -4.14 | **.000** | .08-.40 | .21 | .184 | -1.77 | .077 | .04-1.19 |
| **Monthly income*** |  |  |  |  |  |  |  |  |  |  |
| <2333 | 1 |  |  |  |  |  |  |  |  |  |
| 2333 - 4583 | 1.34 | .211 | 1.84 | .066 | .98-1.82 | 1.28 | 1.030 | .30 | .761 | .26-6.20 |
| >4583 | 1.99 | .685 | 2.00 | **.046** | 1.01-3.90 | .54 | .379 | -.88 | .381 | .14-2.14 |
| **Working setting** |  |  |  |  |  |  |  |  |  |  |
| Mostly in presence/in presence | 1 |  |  |  |  |  |  |  |  |  |
| Mostly home working/home working | 1.37 | .212 | .01 | **.045** | 1.01 - 1.85 |  |  |  |  |  |
| **Sentimental status** |  |  |  |  |  |  |  |  |  |  |
| Not single | 1 |  |  |  |  | 1 |  |  |  |  |
| Single | .73 | .100 | -2.32 | **.021** | 0.55-0.95 | .49 | .097 | -3.61 | **.000** | .33-.72 |
| **Being on a diet** |  |  |  |  |  |  |  |  |  |  |
| No | 1 |  |  |  |  | 1 |  |  |  |  |
| Yes | .99 | .125 | -0.10 | .922 | 0.77-1.27 | 1.26 | .289 | 1.00 | .317 | .80-1.97 |
| **Medi-Lite score** | 1.01 | .025 | 0.47 | .642 | 0.96-1.06 | .89 | .038 | -2.79 | **.005** | .81-.96 |
| **Physical activity** |  |  |  |  |  |  |  |  |  |  |
| <2 times/week | 1 |  |  |  |  | 1 |  |  |  |  |
| ≥2 times/week | .98 | .113 | -0.21 | .835 | 0.78-1.22 | .99 | .217 | -.04 | .967 | .64-1.52 |
| **Screen time while eating** |  |  |  |  |  |  |  |  |  |  |
| Never/seldom | 1 |  |  |  |  | 1 |  |  |  |  |
| Often/always | 1.01 | .119 | 0.11 | .910 | 1.05-2.55 | 2.76 | .587 | 4.78 | **.000** | 1.82-4.19 |
| **Smoke habit** |  |  |  |  |  |  |  |  |  |  |
| Never | 1 |  |  |  |  | 1 |  |  |  |  |
| Occasionally | 1.63 | .372 | 2.16 | **.031** | 1.05-2.55 | 1.68 | .552 | 1.58 | .114 | .88-3.20 |
| Everyday | 1.93 | .342 | 3.72 | **.000** | 1.37-2.73 | 2.94 | 1.010 | 3.14 | **.002** | 1.50-5.77 |
| **BMI** | 1.03 | .012 | 2.32 | **.020** | 1.00-1.05 | 1.07 | .030 | 2.40 | **.016** | 1.01-1.13 |
| **WHO-5** |  |  |  |  |  |  |  |  |  |  |
| Not at risk | 1 |  |  |  |  | 1 |  |  |  |  |
| At risk | 1.41 | .167 | 2.92 | **.003** | 1.12-1.78 | 1.04 | .204 | .22 | .827 | .71-1.53 |
| **SILS-ITA** |  |  |  |  |  |  |  |  |  |  |
| Good HL | 1 |  |  |  |  | 1 |  |  |  |  |
| Bad HL | .76 | .136 | -1.53 | .126 | .53-1.08 | .58 | .151 | -2.08 | .**037** | .35-0.97 |
| **WLB score** | 1.01 | .015 | .55 | .582 | .98-1.04 |  |  |  |  |  |
| **Job satisfaction** |  |  |  |  |  |  |  |  |  |  |
| Satisfied | 1 |  |  |  |  |  |  |  |  |  |
| Unsatisfied | .78 | .097 | -2.03 | **.043** | .61-.99 |  |  |  |  |  |
| **IBS score** | 1.05 | .011 | 4.89 | **.000** | 1.03-1.07 | 1.09 | .024 | 3.85 | **.000** | 1.04-1.14 |

Abbreviations: OR, Odds Ratio; SE, Standard Error; CI, Confidence Interval; Medi-Lite: Literature-based adherence score to Mediterranean diet; BMI, Body Mass Index; WHO-5, 5-item World Health Organization Well-Being Index questionnaire; SILS-ITA, Italian Single-Item Literacy Screener; HL, Health Literacy; WLB, Work Life Balance; IBS, Impulse Buying Scale. In order to enhance readability, p-values < 0.05 are shown bolded.

*currency: euros

# Supplementary Material 1.3 - Sample of DELIvery Choice In OUr Society (DELICIOUS) questionnaire.

**Personal data and general information**

1. Gender:
   1. a. Male
   2. b. Female
   3. c. Non binary/other
2. Age: _____
3. City of residence: ____________
4. Nationality:
   1. Italian
   2. Other: ________
5. Sentimental status:
   1. Single
   2. In a relationship
   3. Married
   4. Divorced
6. Do you live with someone?
   1. No, I live alone
   2. I live with my family
   3. I live with my partner
   4. I live with my housemate
   5. Other: __________
7. What is your individual monthly income in euros?
   1. < 1250
   2. 1250 – 2333
   3. 2334 – 4583
   4. 4584 – 6250
   5. > 6250
8. What is the highest degree of education you achieved?
   1. None
   2. Primary school
   3. Secondary school
   4. Bachelor degree
   5. PhD, Master degree or postdoc
9. What kind of work do you do?
   1. Unemployed
   2. Homemaker
   3. Retired
   4. Student
   5. Student worker
   6. Employee
   7. Self-employee/Freelance worker
   8. Other
10. What is your work setting?
    1. Full presence
    2. Full smart working
    3. Mostly smart working
    4. Mostly presence
11. Have you smoked at least 100 cigarettes in your life (5 packs)?
    1. Yes
    2. No
12. Do you smoke?
    1. Yes, on a daily basis
    2. Yes, occasionally
    3. No
13. Conditional question: If you answer “Yes” to question 13 and “No” to question 14:
    How long ago did you quit smoking?
    1. < 6 months
    2. > 6 months

**Physical activity**

1. How tall are you? (in cm) __________
2. How much do you weigh? __________
3. During the last week, on how many days did you get at least 30 minutes of physical activity that made you breathe hard for at least 30 minutes? (This includes sports, exercises, walking, cycling for exercise, or moving between different places. It does not include housework or physical activity that is part of your job): __________
4. Are you on a diet?
   1. No
   2. Yes

**Food delivery usage habits**

1. Have you used Online Food Delivery (OFD) services in the last 12 months?
   1. Yes
   2. No → Go to next section
2. For each of the five statements, please give the answer that comes closest to how you have felt over the past two weeks. Higher numbers correspond to greater well-being. For example: If you felt happy and in a good mood more than half the time in the last two weeks, please tick the box with the number 3 in the top right-hand corner.
   0 = At no time, 1 = Some of the time, 2 = Less than half the time, 3 = More than half the time, 4 = Most of the time, 5 = Always

| **In the last two weeks** | **0** | **1** | **2** | **3** | **4** | **5** |
| --- | --- | --- | --- | --- | --- | --- |
| I felt cheerful and in good spirits. |  |  |  |  |  |  |
| I felt calm and relaxed. |  |  |  |  |  |  |
| I felt active and vigorous. |  |  |  |  |  |  |
| I woke up feeling fresh and rested. |  |  |  |  |  |  |
| My daily life has been filled with things that interest me. |  |  |  |  |  |  |

1. For each of the following five statements, please indicate the answer that best describes your condition:

|  | **1** | **2** | **3** | **4** | **5** | **6** | **7** |
| --- | --- | --- | --- | --- | --- | --- | --- |
|  | **Very rarely** | | **Sometimes** | | | **Very often** | |
| When I go shopping, I buy things that I did not intend to purchase. |  |  |  |  |  |  |  |
| I am a person who makes unplanned purchases. |  |  |  |  |  |  |  |
|  | **Strongly disagree** | | **Neither** | | | **Strongly agree** | |
| When I see something that really interests me, I buy it without considering the consequences. |  |  |  |  |  |  |  |
| It is fun to buy spontaneously. |  |  |  |  |  |  |  |
| I avoid buying things that are not on my shopping list. (Reverse Coded) |  |  |  |  |  |  |  |

1. How often do you need to have someone help you when you read instructions, pamphlets, or other written material from your doctor or pharmacy?
   1. Never;
   2. Rarely;
   3. Sometimes;
   4. Often;
   5. Always.
2. I usually watch something on PC/TV/mobile phone while eating:
   1. Never or rarely
   2. Often or always
3. What is the consumption of the following food groups?
   1. FRUIT: 1 portion: 150 g
      1. <1 portion/die
      2. 1-2 portion/die
      3. >2 portion/die
   2. VEGETABLES 1 portion: 100 g
      1. <1 portion/die
      2. 1-2.5 portion/die
      3. >2.5 portion/die
   3. LEGUMES 1 portion: 70 g
      1. <1 portion/die
      2. 1-2 portion/die
      3. >2 portion/die
   4. CEREALS 1 portion: 130 g
      1. <1 portion/die
      2. 1-1.5 portion/die
      3. >1.5 portion/die
   5. FISH 1 portion: 100 g
      1. <1 portion/die
      2. 1-2.5 portion/die
      3. >2.5 portion/die
   6. MEAT AND MEAT PRODUCTS 1 portion: 80 g
      1. <1 portion/die
      2. 1-1.5 portion/die
      3. >1.5 portion/die
   7. DAIRY PRODUCTS 1 portion: 180 g
      1. <1 portion/die
      2. 1-1.5 portion/die
      3. >1.5 portion/die
   8. ALCOHOL 1 Alcohol Unit (AU) = 12 g
      1. <1 AU/die
      2. 1-2 AU/die
      3. >2 AU/die
   9. OLIVE OIL
      1. Occasional use
      2. Frequent use
      3. Regular use

**Additional section for workers only (respondents who provided answers e/f/g to question 9)**

1. When I reﬂect over my work and non-work activities (your regular activities outside of work such as family, friends, sports, study, etc.), over the past three months, I conclude that:
   1 = Strongly disagree; 2 = Disagree; 3 = Undecided; 4 = Agree; 5 = Strongly agree

|  | 1 | 2 | 3 | 4 | 5 |
| --- | --- | --- | --- | --- | --- |
| I currently have a good balance between the time I spend at work and the time I have available for non-work activities. |  |  |  |  |  |
| I have difﬁculty balancing my work and non-work activities. |  |  |  |  |  |
| I feel that the balance between my work demands and non-work activities is currently about right. |  |  |  |  |  |
| Overall, I believe that my work and non-work life are balanced. |  |  |  |  |  |

1. Taking everything into consideration, how do you feel about your job as a whole?
   1. 1 = I am extremely dissatisfied;
   2. 2 = I am very dissatisfied;
   3. 3 = I am quite dissatisfied;
   4. 4 = I am indifferent;
   5. 5 = I am quite satisfied;
   6. 6 = I am very satisfied;
   7. 7 = I am extremely satisfied.
